# The Effect of Different Storage Media on Color Stability of Self-Adhesive Composite Resin Cements for up to One Year

**DOI:** 10.3390/ma10030300

**Published:** 2017-03-16

**Authors:** Anja Liebermann, Malgorzata Roos, Bogna Stawarczyk

**Affiliations:** 1 Department of Prosthodontics, Dental School, Ludwig-Maximilians-University Munich, Goethestrasse 70, 80336 Munich, Germany; bogna.stawarczyk@med.uni-muenchen.de; 2 Department of Biostatistics at Epidemiology, Biostatistics and Prevention Institute, University of Zurich, Hirschgraben 84, 8001 Zurich, Switzerland; mroos@ifspm.uzh.ch

**Keywords:** self-adhesive composite resin cements, curry solution, cress solution, red wine, distilled water

## Abstract

The aim of this study was to analyze the long-term color stability of eight self-adhesive composite resin cements (SACRCs) after storage in diverse media for up to one year. 480 discs (diameter: 12 mm/thickness: 1.0 ± 0.05 mm) were fabricated (n = 60/SACRC): (1) BeautyCem (BEA); (2) Bifix SE (BIF); (3) Clearfil SA Cement Automix (CLE); (4) RelyX Unicem 2 Automix (RXU); (5) SeT (SET); (6) SmartCem 2 (SMC); (7) SoloCem (SOC); and (8) SpeedCEM (SPC). After polishing, specimens were immersed in (a) red wine (RW); (b) curry-solution (CU); (c) cress-solution (CR); and (d) distilled water (DW) at 37 °C and measured after 7, 28, 90, 180, and 365 days for color differences (ΔE) and water absorption (WA). Non-aged specimens were used as baselines. After 365 days, all of the discs were polished and their ΔE was measured. Data were analyzed using Kolmogorov-Smirnov, partial-eta-squared/η_P_^2^, 3-/1-way ANOVA with Tukey-HSD post-hoc test (α = 0.05). Significant differences occurred between all SACRCs for WA (*p* ≤ 0.003), except in RXU and in SET and in ΔE (*p* ≤ 0.002), except in SET and SPC. The significantly highest WA presented in SOC; the lowest showed in BEA. Significant ΔE differences and a decrease after polishing between all storage media were found (*p* < 0.001) with highest values for RW, followed by CU, CR, and DW. The lowest ΔE was measured for CLE, followed by SOC, BIF, RXU, BEA, SPC, SET, and SMC (*p* < 0.001) and increased significantly during aging. The highest ΔE decrease presented in BEA. SACRCs showed an increase in WA/ΔE within total aging time. Discoloration could not be removed completely by polishing. SACRCs need to be carefully selected for restorations in the esthetical zone with visible restoration margins. Polishing can significantly reduce the marginal discoloration.

## 1. Introduction

Patients’ esthetic demands for the highest quality tooth-colored restorative solutions that have the best natural appearance are steadily increasing. At the same time, restorative dental materials and the adhesive cements such as self-adhesive composite resin cements (SACRCs) are subjected to major effects resulting from varying liquids and temperatures during food intake. It is already known that the continuous exposure of the restoration margins to the oral environment reduces the mechanical properties by hydrolytic degradation [1,2]. This leads to sorption that may result in swelling of the material, weakening of the polymer network, degradation of the filler matrix composite and, consequently, to secondary caries and hypersensitivity of the teeth at restoration margins [3,4,5,6]. Apart from the mechanical properties, the embedding of color particles from food may also lead to adverse visual effects with discoloration. Higher sorption appears with increasing storage time, while being unaffected by the storage medium [7,8]. When reviewing all fixing composite resins, the interactions with humidity appear to be higher with composite luting materials, such as SACRCs, than for conventional cements [9]. In spite of this, SACRCs are used very commonly in dental practice to insert restorations due to their positive aspects. The use of SACRCs constitutes a time-saving type of fixing that is less technique sensitive and very simple. SACRCs do not require pretreatment of the hard tooth tissues with adhesive systems [10,11]. Compared to conventional composite resins, SACRCs exhibit almost identical compositions in respect of the functional monomers, such as bisphenol-A-diglycidylmethacrylate (Bis-GMA), urethandimethacrylate (UDMA), and hydroxyethylmethacrylate (HEMA), as well as the filler content. However, they contain additional acidic groups (for example, phosphoric acid esters or carboxylate groups). These lead to an improved adhesion to the hard tooth tissues by demineralization of the tooth stump, in contrast to the purely micromechanical adhesion [10,12,13,14,15], which is important for a tight marginal seal. A permanent, stable marginal seal without micro-gaps and stability of the color is a key factor for the long-term success of a restoration and is one of the criteria for the currently much used SACRCs. The aforementioned sorption and the potentially resulting micro-gaps, due to liberation of filler particles, may lead to discoloration of the margins, which is, alongside secondary caries, one of the most common causes for a necessary renewal of a restoration [16,17,18,19,20,21,22,23,24,25]. Soluble color molecules with electrostatic charges consequently move into the lower layers and remain there causing discolorations [3,7,26,27]. These discolorations can be differentiated as extrinsic and intrinsic discolorations. Extrinsic discolorations are the surface agglomeration of plaque and food particles. They can be fully removed by cleaning and polishing as is performed in oral hygiene treatments [28,29]. Intrinsic discoloration, however, is a deeper-reaching discoloration, where dye components enter the deeper layers of polymer-based materials for example, which cannot be removed by polishing [30]. This leads to permanent discoloration of the materials, additionally increasing with higher surface roughness [31,32,33]. A rate of discoloration (ΔE value) below 3.3, by a spectrophotometer measurement, is generally specified as clinically not detectable [34]. The medium red wine seems to have the highest discoloration rates after short term storage [35,36,37,38].

To date, there is limited information available about the discoloration potential of different SARCs, especially in comparing diverse brands. The aim of this study was to compare the sorption and color stability of 8 SACRCs for one year of storage in the 4 media: red wine, curry solution, cress solution, and distilled water.

The tested null hypotheses were:

1. all SACRCs showed no impact of storage medium and aging level on sorption,
2. all SACRCs showed no impact of storage medium and aging level on ∆E, and
3. ∆E/Discoloration rates can be completely removed after final polishing after 365 days storage.

## 2. Material and Methods

### 2.1. Specimens’ Fabrication

Four hundred and eighty standardized discs with a diameter of 12 mm and a thickness of 1.0 ± 0.05 mm were fabricated of eight different SACRCs available on the dental market, all are listed in Table 1.

Each SACRC was slowly filled in a standardized silicon mold (silicon: Heraform RS, type A and B, dark green and white, Heraeus Kulzer, Hanau, Germany) for disk manufacturing and consequently light cured (Elipar S10, 3M, Seefeld, Germany) in overlapping circles according to DIN EN ISO 4049:2010-03 with an exposure distance of 1.5 mm. All fabricated discs were mechanically polished according to a previous study [8]. Each disc specimen was equipped with a precise borehole in the middle of the edge to fix them on a fine wire, positioned upside down in a light-curing resin, to ensure a complete wetting with the different storage media in separate boxes. The color of each specimen was immediately determined, after the specimen´s fabrication and before immersion, with a spectrophotometer (Lambda 35 Perkin Elmer, Perkin Elmer Inc., Waltham, MA, USA) and acted as the baseline for the longitudinal measurements of the discoloration. Afterwards, the storage in a lightproof box of each group was performed at 37 °C in an incubator (HERA cell 150, Thermo scientific, Heraeus Kulzer, Hanau, Germany) and the specimens of each group were randomly divided into 4 sub-groups per 4 storage media (*n* = 15 per medium and SACRC):

(a) *Red wine/RW:* Rioja Cepa Lebrel Joven (Spain) 2013 (pH = 3.8);
(b) *Curry solution/CU:* 40 g curry powder was boiled up with 1 liter of water for 10 min and is filtrated through a fine tea strainer (Ostmann, Dissen a.T.W., Germany) (pH = 5.9);
(c) *Cress solution/CR:* 174 g tamped fresh cress was boiled up in 1 liter of water for 10 min and is afterwards filtrated through a fine tea strainer (pH = 6.0);
(d) *Distilled water/DW:* Aqua Bidest. Kerndl (Weissenfeld, Germany) (pH = 6.7).

Each medium was changed every 14 days. Discoloration rates and sorption (according to weight differences) were measured initially (0 days for baseline) and after 7, 28, 90, 180, and 365 days, as well as after 365 days after polishing, for 1 min (SuperPolish, Kerr Dental, Rastatt, Germany) on both sides. All fabrication and measuring steps were performed through one examiner and instruments were calibrated before analyzing.

### 2.2. Sorption Measurements

All specimens were stored directly after fabrication in a drying chamber (Memmert U30 type with Roth Silica Gel Orange, Carl Roth GmbH + Co. KG, Karlsruhe, Germany) at constant 37 °C until a constant weight was detectable. The drying values by use of the specimens’ weight were measured with the help of a high-precision scale (NewClassic MF Model, MS 104S/M01, Mettler Toledo, Giessen, Germany). The drying duration to achieve this constant weight was approximately 2 weeks with a daily investigation. Sorption was generally performed based on weight differences, which was analyzed with the high-precision scale initially and after storage for 7, 28, 90, 180, and 365 days in the defined test medium. Therefore, specimens were gently wiped with a dry filter paper on both sides before weight and color measurements. Sorption was analyzed with the following formula:

*S* = *m*1 − *m*2

*m*1: specimen´s weight on specific aging level; *m*2: specimen´s first weight of dried condition (initial).

### 2.3. Discoloration Measurements

The discoloration measurements were analyzed initially after fabrication to perform a baseline and after 7, 28, 90, 180, and, 365 days, as well as after 365 days after polishing, using a spectrophotometer (Lambda 35 Perkin Elmer, Perkin Elmer Inc., Waltham, MA, USA). Quantitative measurements were performed with the definite transmission of light through each specimen (wavelength varying between 700 to 400 nm for visible light measurements) at different testing times. The parameters whiteness-blackness/brightness (L*), red-green axis (a*), and yellow-blue axis (b*) were processed in front of a standardized white background. The transmission (illuminant D65) was calculated and measured using the UV WinLabTM 2.8 Software program (Perkin Elmer Inc., Waltham, MA, USA) and the ΔE was analyzed using the Color Application Software V1.00 (Perkin Elmer Inc., Waltham, MA, USA) according to the following formula with respect to Euclidean distance:

$$\Delta E=\sqrt{{\Delta a}^{2}+{\Delta b}^{2}+{\Delta L}^{2}}$$

ΔE: difference in color change; Δa: difference in color change for the red-green axis; Δb: difference in color change in the yellow-blue-axis; ΔL: difference in whiteness-blackness/brightness).

### 2.4. Statistical Analyses

For power analysis, 15 specimens of Clearfil SA Cement (Kuraray Medical Inc. Sakazu, Kurashiki, Okayama, Japan) were fabricated and color as well as water sorption were measured as a baseline. The specimens were stored in red wine for 7 days and the color water sorption was measured again. The computed difference of 5 with SD = 2 was assumed to be relevant; applying the two-group *t*-test with a Bonferroni corrected significance level equal to 0.008. The power analysis, using the software nQuery Advisior (Version 6.0, Statistical Solutions, Saugaus, MA, USA), was performed to determine the optimal sample size, which was 13 per group with a power of 99%. All data were analyzed with the statistical software SPSS Version 23 (SPSS INC, Chicago, IL, USA). Descriptive statistics for all groups were computed and the normality of data distribution was tested using the Kolmogorov-Smirnov test. Higher partial eta squared (η_P_^2^) values indicated higher amounts of variability explained by the variable. Data were analyzed with the 3- and 1-way ANOVA with the Tukey-HSD post-hoc test. Results of statistical analyses, with *p*-values smaller than 0.05, were interpreted as statistically significant.

## 3. Results

According to the Kolmogorov-Smirnov test, 87% of all data were normally distributed and therefore parametric tests were performed.

### 3.1. Impact of Sorption on SACRCs Material/Storage Medium/Aging Level

Descriptive statistics with mean and SD for the sorption was summarized in Table 2.

Within sorption results, SACRCs material exerted the highest influence (*p* < 0.001, η_P_^2^ = 0.886), followed by aging level (*p* < 0.001, η_P_^2^ = 0.101), and storage medium (*p* < 0.001, η_P_^2^ = 0.009). Significant differences occurred between all SACRCs materials (*p* ≤ 0.003), except between RXU and SET (*p* > 0.999). Significantly the highest sorption was presented in SOC, followed by SPC, SMC, BEA, SET, and RXU. The lowest sorption showed in BIF and CLE (Table 3).

Sorption showed significantly higher results for specimens stored in distilled water than for SACRCs stored in cress solution (*p* = 0.023), red wine (*p* < 0.001), or curry solution (*p* < 0.001). No significant differences were analyzed between SACRCs stored in red wine, curry, and cress solution. After storage, an increase in sorption values compared to non-aged specimens was observed, based on the number of storage days (*p* < 0.001). Furthermore, significant differences occurred between 7 storage days and 28, 90, 180, and 365 days (*p* ≤ 0.034) as well as between level 365 storage days and 28, 90, and 180 days (*p* ≤ 0.005).

### 3.2. Impact of Discoloration on SACRCs/Storage Medium/Aging Level

Descriptive statistics with mean and standard deviation (SD) of ΔE of all tested SACRCs for each storage medium and aging level were listed in Table 4.

**Figure 1:**
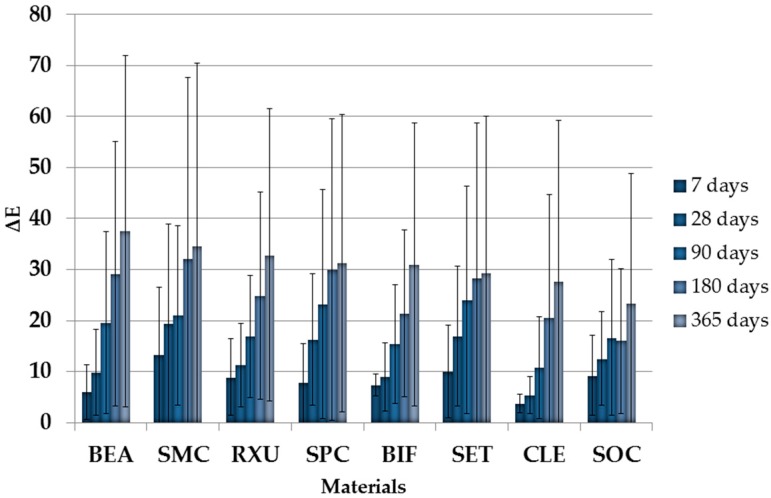
ΔE values of all tested SACRCs over the total aging time of one year (365 days) with pooled storage media in descending order according to day 365.

The storage medium exerted the highest influence on ΔE values (*p* < 0.001; η_P_^2^ = 0.957), followed by the aging level (*p* < 0.001; η_P_^2^ = 0.871), and the SACRCs material (*p* < 0.001; η_P_^2^ = 0.522). Significant differences in ΔE values between all storage media were found (*p* < 0.001). Highest ΔE values were observed for SACRCs stored in red wine, followed by curry solution, cress solution, and distilled water. The significantly lowest ΔE was generally measured for CLE, followed by SOC, BIF, RXU, BEA, SPC, SET, and SMC (all *p* < 0.001). Significant differences occurred between all tested SACRCs (*p* ≤ 0.002), with the exception between SET and SPC (*p* > 0.999). ΔE increased significantly during aging level (Figure 1).

Therefore, significant differences were found between all aging levels with the lowest ΔE values for 7 storage days, followed by aging 28, 90, 180, and 365 days.

### 3.3. Impact of Final Polishing on SACRCs/Storage Medium

**Figure 2:**
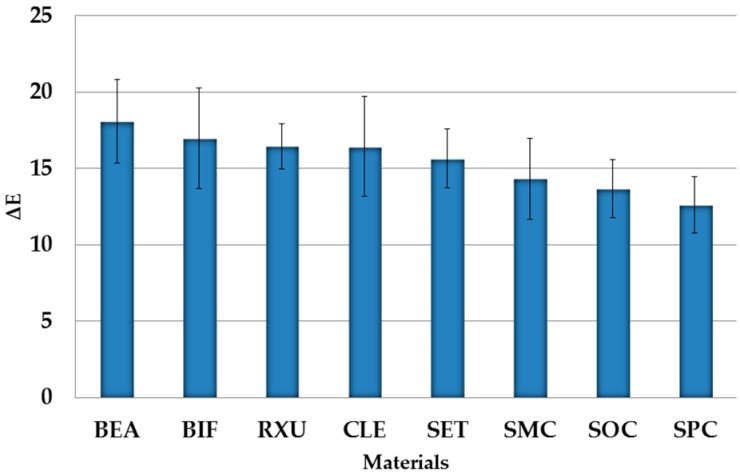
ΔE values after final polishing of each SACRCs material separately with pooled storage media in descending order.

The final polishing showed the highest influence by storage medium (*p* < 0.001, η_P_^2^ = 0.978) followed by SACRCs material (*p* < 0.001, η_P_^2^ = 0.608) on ΔE differences. Final ΔE results, of all SACRCs within the diverse media after polishing, are presented in Table 5 and Figure 2.

The significantly highest ΔE decrease was presented by BEA, followed by SMC, SPC, RXU, BIF, SET, CLE, and SOC. No significant differences occurred between SMC and SPC (*p* = 0.131), BIF and SET (*p* = 0.999), or between CLE and SOC (*p* = 0.136). In addition, the highest ΔE decrease (removal on discoloration) was shown by specimens stored in red wine, followed by curry solution, cress solution, and distilled water, with significant differences between all storage media (*p* < 0.001). With distilled water, the ΔE values of BEA, CLE, SET, and SOC were significantly different (*p* < 0.001) to the measured values of day 365. Further significant differences (*p* < 0.001) were analyzed for red wine stored SACRCs as well as for BEA, BIF, RXU, SET, SMC, SOC, and SPC stored in curry solution (*p* ≤ 0.036). In addition, BEA, SET, and SPC showed significant differences (*p* < 0.001) within specimens stored in cress solution (Table 6).

## 4. Discussion

Margin discolorations of restorations may significantly affect the visual appearance in a negative way, particularly for visible restoration margins in the esthetic zone. In these cases, the type and chemical composition of the composite resins plays an important role. In addition, it depends on the restoration accuracy, the cementation technique and the location of the restoration margin. Tooth-colored restorations are mainly used with conventional or self-adhesive composite resins. Due to the acidic carboxyl or phosphate groups, SACRCs generally exhibit more hydrophilic properties than the non-self-adhesive fixing composites [14,15]. In these cases, one also needs to review the filler particle size, filler particle content, and the filler type, which all significantly contribute to the sorption and solubility [20,21,22,23,24]. In SACRCs the number of organic filler particle matrices decreases in line with the increase in filler particle size. Discolorations by the sorption of Bis-GMA-containing fixing composite resins, like SACRCS, should generally be higher than in materials containing UDMA. The UDMA monomer is said to be less hydrophilic and more resistant to discoloration [22]. The sorption is consequently higher due to the OH groups in the Bis-GMA monomer, which is also able to transport the dye components into a deeper surface layer [17,26,27]. Few conclusions could be drawn from these results because the exact compositions of some of the SACRCs investigated were not available in detail; the aforementioned statements could not be confirmed with the present material information. BIF, which contains both monomers, exhibited the lowest sorption, followed by CLE with the Bis-GMA monomer. In contrast, the SOC containing UDMA had the highest sorption over the one year storage period. In general, the SACRCs continued to absorb water during the storage period, which can be interpreted from the significant differences between the storage times. Based on the significantly different values between day 180 and day 365, there was evidence of further sorption, even after 180 days of storage. In addition, it was found that the sorption differed significantly with all individual materials, apart from RXU and SET. Presumably, this is due to the small differences in the matrix composition and the difference in the content and size of the filler particles. In this case, further studies that could access the exact filler particle sizes and matrix compositions are necessary. Correlation analyses were not possible due to the lack of some information on the materials. There were also significant differences between the storage media. The difference in polarity and particularly in the pH value of the medium might have been the key factor. In one of the following paragraphs, the pH value will be reviewed in more detail. In this study, the sorption was generally below the permitted ISO standard threshold of 40 µg/mm^3^ in all SACRCs tested. This value, which is listed in the ISO standard for storage of 14 days, was not reached, even after one year of permanent storage in the aging medium. Importantly, the permanent storage in the liquids does not exactly represent the intraoral situation, considering that during sleep, or in general with patients who breathe a lot through the mouth, the oral cavity often becomes dry. Furthermore, the time of intraoral food and beverages while eating is reduced due to fast swallowing. The first null hypothesis is rejected based on the present results, because both the storage medium and the storage time exhibited a significant effect on the sorption with all SACRCs.

Since the discoloration results from sorption and embedding of dye particles in the matrix, the SACRCs with larger filler particles and higher filler particle numbers should express more noticeable discoloration [19,23]. The filler particle type itself also appears to have a significant effect on the discoloration rate. These rates were analyzed in this aforementioned study in composite resin artificial teeth and are not directly comparable [25]. Furthermore, these results could not be confirmed in this study, because the smallest ΔE values could be analyzed for CLE (66 wt % filler) and BIF (70 wt % filler). In contrast, SMC (69 wt % filler) and SET (65 wt % filler) exhibited the highest rate of discoloration, even though they had similar filler particle sizes. The storage medium itself also had a significant effect on the rate of discoloration of the SACRCs in the present study. This result could also be confirmed for other artificial materials in further studies [25]. There is also a significant difference between the rates of discoloration of the four media. The highest discoloration in this study was exhibited in red wine, followed by curry solution, cress solution and distilled water. Evidence for the high rate of discoloration for red wine has been found in the past [35,36], while none of the studies known to the authors shows a storage period of one year. Compared to a study that investigated discoloration rates of three different veneering composites over 180 days of storage in three discoloring food medium, only the values for red wine can be considered. With ΔE values between 23.02 and 30.30, they were significantly below the ΔE values of 37.53 to 88.04 for the 180 days measurement in this study. This is presumably due to the SACRC materials flow out of the respective cartridges with small bubbles, or the hydrophilic acidic carboxyl and phosphate groups, otherwise they have very similar compositions. The micro pores remain in the material after photo-curing, and presumably the color particles are better able to embed themselves, which leads to higher ΔE values. In this respect, a material that is free of bubbles would be desirable, and would presumably significantly change the results. Compared to the results of the present study, one could also create a potential link to the different pH levels of the medium. Contrary to the ΔE values, red wine showed the lowest pH level, followed by curry solution, cress solution and distilled water. A lower pH level might negatively affect the surface structure and roughness of the materials [5]. The reduced pH level with red wine is caused by the acids it contains, for example, acetic acid, butyric acid, and lactic acid. Acetic acid can attack polymer material surfaces and thus promote the embedding of color molecules. The alcohol it contains also negatively affects the polymer matrix [37]. The different pH levels of the storage medium did not influence the ΔE values of the resin materials themselves [38]. Surface roughness that might be caused by acidic pH levels increases the susceptibility for extrinsic and intrinsic discolorations of dental restorations [31,32,33]. The SACRCs exhibited significantly different ΔE levels, apart from those between SPC and SET. In general, the lowest discoloration was shown with CLE, and the highest with SMC. No correlation could be drawn to the SACRC compositions. All ΔE levels very much depended on the medium and the storage time that was measured. With each measurement time the ΔE levels significantly increased. These results were already confirmed in previous studies [35,36]. Therefore, the second null hypothesis is also rejected, because both the storage medium and the storage time exhibited a significant effect on the ΔE levels with all SACRCs. 

All measurements were performed using a spectrophotometer in vitro. In Vivo investigations are not possible in this way, because fixing composites resins are subject to constantly changing conditions with the rinsing effect of saliva and varying food products, as well as temperature variations. Moreover, it is not easily possible to remove the materials and automatically analyze them. Only mostly visual or purely subjective measurement methods can be used, which makes it more difficult to compare different materials, and makes it difficult to reproduce. In the present study, the specimens´ surfaces were polished to a high gloss finish. It will not be possible to reproduce this with cervical restoration margins in vivo. The surface is presumably more significantly roughened and could discolor more. In this case, a repolish would be necessary in case of clinically visible discolorations. Clinicians, however, should be aware, that every polishing or repolishing procedure might cause abrasion to the restoration material, the luting materials, as well as the tooth structure itself. Moreover, the specimens were stored for 24 h in the food medium, without any interim cleaning. This does not correspond to the natural conditions, because oral hygiene is generally performed twice daily, which constitutes a type of polishing. This surface cleaning would almost entirely remove extrinsically attached color particles and food components and counteract an intrinsic discoloration [29], except for less accessible approximal areas. The important esthetic appearance of the margins of inlays, partial crowns and full crowns can be restored by polishing [28]. The SACRCs should reveal more slowly occurring discolorations under natural conditions than the specimens stored in this study. Restorations may then feature a ΔE value below 3.3 for a longer period, and therefore appear clinically acceptable [34]. In this study, even the storage in distilled water reached the value of at least 4.4 (SET) for SACRCs before polishing after 365 days, which therefore cannot be regarded as clinically acceptable either. In all materials and storage media, the ΔE levels were between 5.36 (cress) and 32.23 (red wine). Consequently, all specimens showed a clinically unacceptable discoloration after 365 days. These facts are not critical, since storage in a medium up to one year is not realistic. The goal of this study was to extremely age these materials to compare them with respect to the discoloration parameter. The pink color of the polish paste was one limitation of the study. In this case, there was an increase of the ΔE levels between day 365 and after the polish, mainly for the distilled water storage medium. This could be explained by the embedding of the color particles into the small pores of the SACRCs. Further studies should use a neutral polish paste. In general, it was possible to significantly reduce the grade of discoloration in all, but most notably in the seriously discolored specimens with red wine and curry solution. It can be assumed that the discolorations are mostly extrinsic. This could also be shown by a significant formation of plaque on the specimens. However, it was not possible to achieve the ΔE starting levels, because intrinsic discolorations occurred. The storage time was 365 days, which constitutes a very long period and is not comparable to the clinical situation. The third hypothesis, which states that the SACRC discolorations can be fully removed back to the baseline level, can be rejected. The main limitation of the present study was that no control group of conventional composite resin was included and compared. This should be further investigated since the SACRCs could be better classified within all composite resins. With a better knowledge about the discoloration behavior with resultant significant differences between some material brands and storage media, the clinician should carefully select the right SACR for cementation procedure. The margin discolorations of restorations especially for visible margins in the esthetic zone are diet-related. A careful management of a perfect fit of the restoration, the right cementation procedure, as well as continuous check-ups of the restoration margins with timely repolishing of visible staining are essential.

## 5. Conclusions

Within the limitations of this in vitro study, the following conclusions can be drawn:

- SARCs showed a significant increase in sorption and discoloration rates within total aging time.
- Significant differences between all SARCs and media tested occurred for sorption and discoloration rates.
- Significantly lowest discoloration rates were measured for CLE, followed by SOC, BIF, and RXU; the highest were analyzed for SMC, followed by SET, SPC, and BEA.
- Highest discoloration rates were analyzed for red wine, followed by curry-solution, cress-solution, and distilled water.
- Discoloration was mostly extrinsic, but could not be removed completely by polishing procedures.

## Figures and Tables

**Table 1:** Summary of products, abbreviations, manufacturers, Lot. No., and material compositions in alphabetical order.

SACRC	Abbrev.	Manufacturer	Lot. No.	Composition
**BeautyCem SA**	BEA	Shofu Inc., Kyoto, Japan	061201	PASTE A: UDMA, Fluoroboroalumina-SG, SG, Reaction initiators PASTE B: UDMA, 2-HEMA, Carboxylic acid monomer, Phosphonic acid monomer, ZiS, initiator and others
**Bifix SE**	BIF	Voco, Cuxhaven, Germany	1329157	BASE: UDMA, GDMA, initatiors, catalyst CATALYST: UDMA, acidic adhesive monomer, Bis-GMA, GDMA, HPMA, BP, 70 wt %/61 vol %
**Clearfil SA Cement Automix**	CLE	Kuraray Medical Inc., Sakazu, Kurashiki, Okayama, Japan	058AAA	PASTE A: MDP, Bis-GMA, TEGDMA, DMA, Ba-Al fluoro-SG, SiO_2_, BP, initiators PASTE B: Bis-GMA, DMA, Ba-Al fluoro-SG, SiO_2_, pigments, 66 wt %/45 vol % fillers
**RelyX Unicem 2 Automix**	RXU	3M, Seefeld, Germany	522135	methacrylated phosphoric esters, methacrylated monomer, DMA fillers, silanated fillers. 72 wt %/54 vol % fillers
**seT**	SET	SDI, Koeln, Germany	S13061003	35 wt % methacrylate ester; 65 wt % inorganic filler
**SmartCem 2**	SMC	Dentsply Detrey, Konstanz, Germany	130430	UDMA, EBPADMA urethane resin, di- and tri-functional diluents, PENTA, 69 wt %/46 vol % fillers
**SoloCem**	SOC	Coltène/Whaledent, Altstaetten, Switzerland	F28793	UDMA, TEGDMA, 4-META, 2-HEMA, DBP; BP
**SpeedCEM**	SPC	Ivoclar Vivadent, Schaan, Liechtenstein	S40661	DMA, YTF, co-polymer, glass filler 40 vol %, SiO_2_, adhesive monomer, initiators, stabilizers and pigments

**Table 2:** Descriptive statistics with mean and standard deviation (SD) of weight analysis (water absorption) of tested SACRCs for each storage medium and aging level in alphabetical order, respectively.

Material	0d	7d	28d	90d	180d	365d
**Distilled water**						
**BEA**	0.337 ± 0.008	0.342 ± 0.007	0.346 ± 0.007	0.347 ± 0.007	0.348 ± 0.007	0.348 ± 0.007
**BIF**	0.366 ± 0.011	0.368 ± 0.011	0.375 ± 0.011	0.375 ± 0.011	0.376 ± 0.011	0.375 ± 0.011
**CLE**	0.353 ± 0.011	0.357 ± 0.011	0.358 ± 0.011	0.360 ± 0.011	0.360 ± 0.011	0.361 ± 0.011
**RXU**	* 0.357 ± 0.008	* 0.363 ± 0.008	* 0.364 ± 0.008	* 0.367 ± 0.008	* 0.368 ± 0.008	0.367 ± 0.008
**SET**	0.356 ± 0.012	0.368 ± 0.013	0.368 ± 0.013	0.369 ± 0.013	0.369 ± 0.013	0.369 ± 0.012
**SMC**	0.388 ± 0.011	0.394 ± 0.011	0.396 ± 0.011	0.396 ± 0.011	0.395 ± 0.011	0.395 ± 0.011
**SOC**	0.431 ± 0.013	0.439 ± 0.014	0.441 ± 0.014	0.442 ± 0.014	0.443 ± 0.014	0.443 ± 0.014
**SPC**	0.411 ± 0.015	0.416 ± 0.015	0.417 ± 0.015	0.417 ± 0.015	0.417 ± 0.015	0.417 ± 0.015
**Red wine**						
**BEA**	0.334 ± 0.011	0.339 ± 0.010	0.342 ± 0.010	0.344 ± 0.010	0.345 ± 0.011	0.351 ± 0.011
**BIF**	0.368 ± 0.012	0.371 ± 0.012	0.376 ± 0.013	0.378 ± 0.012	0.378 ± 0.012	0.394 ± 0.015
**CLE**	0.357 ± 0.009	0.361 ± 0.009	0.361 ± 0.010	0.365 ± 0.009	0.367 ± 0.100	0.373 ± 0.011
**RXU**	0.359 ± 0.008	0.363 ± 0.008	0.365 ± 0.008	0.367 ± 0.008	0.369 ± 0.007	0.382 ± 0.013
**SET**	* 0.350 ± 0.016	0.361 ± 0.016	* 0.362 ± 0.016	0.362 ± 0.016	0.360 ± 0.015	0.363 ± 0.016
**SMC**	0.383 ± 0.008	0.391 ± 0.008	0.392 ± 0.008	0.393 ± 0.008	0.394 ± 0.008	0.406 ± 0.013
**SOC**	0.440 ± 0.016	0.447 ± 0.016	0.450 ± 0.0160	0.451 ± 0.016	0.452 ± 0.016	* 0.467 ± 0.025
**SPC**	0.412 ± 0.016	0.417 ± 0.016	0.417 ± 0.016	0.419 ± 0.016	0.423 ± 0.016	0.433 ± 0.016
**Curry solution**						
**BEA**	0.337 ± 0.009	0.342 ± 0.009	0.346 ± 0.010	0.347 ± 0.010	0.347 ± 0.010	0.347 ± 0.010
**BIF**	0.370 ± 0.010	* 0.371 ± 0.010	0.379 ± 0.010	* 0.380 ± 0.011	* 0.380 ± 0.010	* 0.380 ± 0.011
**CLE**	0.358 ± 0.007	0.362 ± 0.007	* 0.364 ± 0.008	* 0.367 ± 0.007	* 0.367 ± 0.007	* 0.367 ± 0.007
**RXU**	0.358 ± 0.009	0.362 ± 0.009	0.367 ± 0.009	0.368 ± 0.009	0.368 ± 0.009	0.369 ± 0.009
**SET**	0.357 ± 0.009	0.369 ± 0.010	0.370 ± 0.01	0.370 ± 0.010	0.369 ± 0.010	0.369 ± 0.010
**SMC**	0.388 ± 0.012	0.394 ± 0.012	0.394 ± 0.012	0.394 ± 0.012	0.393 ± 0.012	0.393 ± 0.012
**SOC**	0.443 ± 0.018	0.450 ± 0.018	0.452 ± 0.019	0.453 ± 0.019	0.454 ± 0.019	0.454 ± 0.018
**SPC**	0.410 ± 0.017	0.416 ± 0.017	0.416 ± 0.017	0.417 ± 0.017	0.416 ± 0.017	0.418 ± 0.016
**Cress solution**						
**BEA**	0.332 ± 0.008	0.338 ± 0.008	0.342 ± 0.008	0.342 ± 0.008	0.343 ± 0.008	0.344 ± 0.008
**BIF**	0.367 ± 0.010	0.370 ± 0.010	0.376 ± 0.011	0.376 ± 0.010	0.376 ± 0.010	0.377 ± 0.010
**CLE**	0.355 ± 0.009	0.360 ± 0.009	0.361 ± 0.010	0.362 ± 0.010	0.363 ± 0.009	0.364 ± 0.010
**RXU**	0.356 ± 0.007	0.360 ± 0.007	0.365 ± 0.008	0.366 ± 0.007	0.366 ± 0.007	0.369 ± 0.006
**SET**	0.360 ± 0.009	0.371 ± 0.010	0.371 ± 0.010	0.370 ± 0.010	0.370 ± 0.010	0.371 ± 0.010
**SMC**	0.390 ± 0.009	0.395 ± 0.004	0.395 ± 0.009	0.395 ± 0.009	0.395 ± 0.009	0.395 ± 0.009
**SOC**	0.448 ± 0.015	0.455 ± 0.015	0.456 ± 0.015	0.457 ± 0.015	0.458 ± 0.015	0.459 ± 0.015
**SPC**	0.408 ± 0.015	0.414 ± 0.015	0.414 ± 0.015	0.414 ± 0.015	0.414 ± 0.015	0.414 ± 0.015

* not normally distributed.

**Table 3:** Weight (in µg) and water absorption (in µg/mm^3^) after 1 year (365 days) of storage with pooled aging level for each storage medium and SACRCs in alphabetical order.

Material	Distilled Water	Red Wine	Curry Solution	Cress Solution	Sorption
**BEA**	345,000 ± 8000	343,000 ± 12,000	344,000 ± 10,000	340,000 ± 9000	26.53
**BIF**	372,000 ± 11,000	377,000 ± 15,000	377,000 ± 11,000	374,000 ± 11,000	8.84
**CLE**	358,000 ± 11,000	364,000 ± 12,000	364,000 ± 8000	361,000 ±10,000	17.68
**RXU**	364,000 ± 9000	368,000 ± 12,000	365,000 ± 10,000	364,000 ± 8000	17.68
**SET**	366,000 ± 13,000	360,000 ± 16,000	368,000 ± 11,000	369,000 ± 10,000	39.79
**SMC**	394,000 ± 11,000	393,000 ± 11,000	393,000 ±12,000	394,000 ± 9000	26.53
**SOC**	440,000 ± 14,000	451,000 ± 19,000	451,000 ± 18,000	455,000 ± 15,000	35.37
**SPC**	416,000 ± 15,000	420,000 ± 17,000	451,000 ± 17,000	413,000 ± 15,000	26.53

**Table 4:** Descriptive statistics with mean and standard deviation (SD) of ΔE of tested SACRCs for each storage medium and aging level (without final polishing values) in alphabetical order, respectively.

Storage Level (Days)	Material	Distilled Water	Red Wine	Curry Solution	Cress Solution
**7**	**BEA**	1.66 ± 0.46 ^a^	6.7 ± 1.32 ^bc^	13.87 ± 3.65 ^b^	1.72 ± 0.64 ^a^
	**BIF**	* 4.73 ± 2.86 ^cd^	* 7.60 ± 2.57 ^c^	* 10.45 ± 1.93 ^ab^	6.77 ± 1.42 ^e^
	**CLE**	2.19 ± 1.00 ^ab^	* 5.06 ± 2.03 ^ab^	5.67 ± 1.73 ^a^	* 1.95 ± 1.36 ^ab^
	**RXU**	3.31 ± 0.74 ^bc^	* 20.37 ± 3.07 ^e^	9.08 ± 2.45 ^ab^	2.91 ± 1.30 ^abc^
	**SET**	1.38 ± 0.78 ^a^	* 12.98 ± 2.45 ^d^	22.55 ± 5.52 ^c^	3.21 ± 0.95 ^bc^
	**SMC**	4.51 ± 0.74 ^cd^	13.12 ± 2.79 ^d^	33.41 ± 11.44 ^d^	1.85 ± 0.39 ^a^
	**SOC**	5.69 ± 1.27 ^cd^	3.92 ± 0.90 ^a^	21.93 ± 2.62 ^c^	5.41 ± 1.81 ^d^
	**SPC**	0.84 ± 0.37 ^a^	6.50 ± 1.30 ^bc^	19.89 ± 5.36 ^c^	3.83 ± 1.06 ^c^
**28**	**BEA**	1.79 ± 0.44 ^a^	22.61 ± 1.53 ^cd^	9.32 ± 1.58 ^a^	* 5.42 ± 4.07 ^bc^
	**BIF**	* 3.30 ± 2.51 ^bc^	13.32 ± 2.14 ^a^	16.90 ± 4.88 ^b^	2.48 ± 1.39 ^a^
	**CLE**	* 2.07 ± 1.03 ^ab^	10.58 ± 2.07 ^a^	6.38 ± 1.93 ^a^	2.53 ± 1.35 ^a^
	**RXU**	* 4.20 ± 1.31 ^cd^	19.34 ± 2.58 ^bc^	18.56 ± 3.95 ^b^	3.16 ± 0.92 ^ab^
	**SET**	1.96 ± 0.53 ^ab^	33.71 ± 2.99 ^e^	* 24.89 ± 4.84 ^c^	7.23 ± 2.46 ^cd^
	**SMC**	3.76 ± 0.58 ^cd^	23.43 ± 4.84 ^d^	47.55 ± 6.66 ^d^	2.77 ± 0.61 ^a^
	**SOC**	4.74 ± 1.28 ^d^	17.22 ± 6.33 ^b^	24.20 ± 2.38 ^c^	4.02 ± 1.22 ^ab^
	**SPC**	1.65 ± 0.48 ^a^	33.36 ± 2.25 ^e^	21.14 ± 2.55 ^bc^	9.17 ± 2.55 ^d^
**90**	**BEA**	2.61 ± 0.50 ^b^	40.24 ± 5.08 ^c^	31.67 ± 4.84 ^d^	3.65 ± 1.09 ^ab^
	**BIF**	* 6.37 ± 2.55 ^d^	21.68 ± 4.31 ^a^	30.09 ± 6.80 ^d^	3.69 ± 1.43 ^ab^
	**CLE**	2.85 ± 1.48 ^b^	25.54 ± 3.00 ^ab^	12.14 ± 2.61 ^a^	2.50 ± 1.31 ^a^
	**RXU**	0.98 ± 0.55 ^a^	30.96 ± 5.21 ^b^	22.97 ± 5.19 ^bc^	12.57 ± 1.04 ^d^
	**SET**	4.59 ± 1.41 ^c^	58.09 ± 3.63 ^d^	23.54 ± 4.43 ^c^	9.81 ± 1.84 ^c^
	**SMC**	6.61 ± 1.03 ^d^	42.85 ± 6.41 ^c^	30.73 ± 7.74 ^d^	3.80 ± 1.07 ^ab^
	**SOC**	3.84 ± 0.84 ^bc^	39.19 ± 8.08 ^c^	18.77 ± 3.07 ^bc^	4.79 ± 1.10 ^b^
	**SPC**	5.35 ± 1.20 ^cd^	58.81 ± 4.10 ^d^	17.54 ± 1.46 ^ab^	11.15 ± 1.51 ^cd^
**180**	**BEA**	5.51 ± 0.74 ^bc^	63.00 ± 6.13 ^d^	41.29 ± 9.81 ^d^	6.97 ± 0.92 ^c^
	**BIF**	* 10.49 ± 2.9 ^d^	43.91 ± 6.95 ^b^	26.45 ± 5.35 ^c^	4.67 ± 1.52 ^ab^
	**CLE**	4.81 ± 1.30 ^ab^	58.69 ± 6.88 ^cd^	15.67 ± 4.89 ^a^	3.20 ± 1.35 ^a^
	**RXU**	* 6.08 ± 1.84 ^bc^	56.50 ± 7.07 ^c^	18.65 ± 1.41 ^ab^	* 18.19 ± 2.27 ^e^
	**SET**	3.21 ± 0.95 ^a^	* 76.30 ± 2.93 ^e^	* 21.34 ± 3.33 ^abc^	12.64 ± 2.00 ^d^
	**SMC**	8.87 ± 1.43 ^d^	88.04 ± 2.00 ^f^	26.37 ± 5.99 ^c^	* 5.42 ± 1.93 ^bc^
	**SOC**	4.37 ± 1.00 ^ab^	37.53 ± 5.70 ^a^	16.53 ± 2.36 ^a^	5.71 ± 0.77 ^bc^
	**SPC**	6.97 ± 1.72 ^c^	76.80 ± 1.22 ^e^	22.85 ± 1.61 ^bc^	13.29 ± 1.96 ^d^
**365**	**BEA**	12.34 ± 1.57 ^c^	91.82 ± 0.63 ^d^	31.26 ± 4.50 ^c^	14.73 ± 1.77 ^b^
	**BIF**	17.85 ± 3.16 ^d^	* 74.97 ± 3.09 ^b^	* 22.76 ± 4.80 ^b^	8.32 ± 1.20 ^a^
	**CLE**	8.66 ± 1.33 ^b^	* 78.62 ± 2.17 ^bc^	15.57 ± 3.84 ^a^	8.08 ± 1.60 ^a^
	**RXU**	13.00 ± 1.69 ^c^	78.97 ± 1.41 ^c^	20.68 ± 1.33 ^b^	18.78 ± 1.97 ^c^
	**SET**	* 4.40 ± 0.77 ^a^	* 78.18 ± 1.29 ^bc^	* 20.28 ± 3.95 ^b^	14.34 ± 2.13 ^b^
	**SMC**	12.31 ± 1.43 ^c^	91.14 ± 0.51 ^d^	28.05 ± 5.03 ^c^	7.01 ± 2.03 ^a^
	**SOC**	6.41 ± 1.39 ^a^	64.17 ± 8.51 ^a^	16.12 ± 1.84 ^a^	6.47 ± 1.68 ^a^
	**SPC**	8.95 ± 1.94 ^b^	77.92 ± 0.78 ^bc^	21.21 ± 1.74 ^b^	16.84 ± 2.05 ^c^

* not-normally distributed; ^abcdef^ Different letters within the same column indicate significant differences between the SARCs (except 365 days polished groups).

**Table 5:** Descriptive statistics with mean and standard deviation (SD) of ΔE of finally polished SACRCs for each storage medium and aging level in alphabetical order.

Material	Distilled Water	Red Wine	Curry Solution	Cress Solution
**BEA**	* 7.64 ± 2.86	* 32.23 ± 3.33	14.85 ± 12.13	5.36 ± 0.72
**BIF**	17.22 ± 3.08	* 25.23 ± 2.95	* 17.84 ± 6.10	* 7.54 ± 1.07
**CLE**	11.17 ± 1.07	27.32 ± 4.52	* 18.24 ± 5.84	8.94 ± 1.63
**RXU**	14.25 ± 1.88	19.16 ± 1.35	14.37 ± 1.19	18.01 ± 1.47
**SET**	6.47 ± 1.36	30.74 ± 2.24	* 14.49 ± 2.50	* 10.86 ± 1.58
**SMC**	12.10 ± 2.33	20.85 ± 3.06	* 17.57 ± 4.34	6.77 ± 0.95
**SOC**	9.52 ± 1.33	23.97 ± 2.28	14.39 ± 2.42	6.81 ± 1.51
**SPC**	7.58 ± 2.19	21.70 ± 2.28	12.31 ± 1.58	8.87 ± 1.36

* not-normally distributed.

**Table 6:** Mean ΔE value differences between polished and final (365 days) values in alphabetical order.

Material	Distilled Water	Red Wine	Curry Solution	Cress Solution
**BEA**	* 4.70 ± 0.84	* 59.58 ± 0.87	* 16.41 ± 3.34	* 9.37 ± 0.49
**BIF**	0.63 ± 1.14	* 49.73 ± 1.10	* 4.92 ± 2.01	0.78 ± 0.42
**CLE**	* −2.51 ± 0.44	* 51.30 ± 1.29	−2.67 ± 1.80	−0.86 ± 0.59
**RXU**	−1.25 ± 0.65	* 59.81 ± 0.50	* 6.31 ± 0.46	0.77 ± 0.63
**SET**	* −2,07 ± 0.40	* 47.44 ± 0.67	* 5.79 ± 1.21	* 3.48 ± 0.69
**SMC**	0.21 ± 0.71	* 70.29 ± 0.80	* 10.48 ± 1.72	0.24 ± 0.58
**SOC**	* −3.10 ± 0.50	* 40.20 ± 2.27	* 1.73 ± 0.79	−0.34 ± 0.58
**SPC**	1.37 ± 0.76	* 56.22 ± 0.62	* 8.89 ± 0.61	* 7.97 ± 0.64

* significant differences between both tested values.

## Abbreviations

The following abbreviations are used in this manuscript:

BEA	BeautyCem
BIF	Bifix SE
CLE	Clearfil SA Cement Automix
RXU	RelyX Unicem 2 Automix
SET	SET (Cement)
SMC	SmartCem 2
SOC	SoloCem
SPC	SpeedCEM
CU	curry-solution
CR	cress-solution
DW	distilled water
RW	red wine
WA	water sorption
SACRC	self-adhesive composite resin cement
UDMA	urethandimethacrylate
HEMA	hydroxyethylmethacrylate
Bis-GMA	bisphenol-A-diglycidylmethacrylate
DMA	dimethacrylate
BP	benzoylperoxide
SG	silicate glass
ZiS	zirconium silicate
YTF	ytterbium trifluoride
DBP	dibenzoyl peroxide
GDMA	glycerol-dimethacrylate
HPMA	hydroxypropylmethacrylate
TEGDMA	triethyleneglycol-dimethacrylate
META	methacryloyloxyethyl-trimellitate-anhydride
Ba-Al	barium-aluminium
SiO_2_	silicon dioxide
EBPADMA	ethoxylated bisphenol A dimethacrylate
PENTA	dipentaerythritol pentaacrylate monophosphate
ΔE	color difference
